# Can magnetic resonance spectroscopy differentiate malignant and benign causes of lymphadenopathy? An *in-vitro* approach

**DOI:** 10.1371/journal.pone.0182169

**Published:** 2017-08-08

**Authors:** Lionel Buré, Louis-Martin Boucher, Miriam Blumenkrantz, Stefan Schob, Pierre Lafaye de Micheaux, Caroline Reinhold, Benoit Gallix

**Affiliations:** 1 Department of Radiology, McGill University Health Center, Montreal General Hospital, Montreal, Qc, Canada; 2 Department of Adult and Pediatric Pathology, McGill University Health Center, Royal Victoria Hospital, Montreal, Qc, Canada; 3 Department of Neuroradiology, Leipzig University Hospital, Leipzig, Germany; 4 School of Mathematics and Statistics, University of New South Wales, Sydney, Australia; National Research Council of Italy, ITALY

## Abstract

Lymphadenopathy continues to be a common problem to radiologists and treating physicians because of the difficulty in confidently categorizing a node as being benign or malignant using standard diagnostic techniques. The goal of our research was to assess whether magnetic resonance (MR) spectroscopy contains the necessary information to allow differentiation of benign from malignant lymph nodes in an *in-vitro* approach using a modern pattern recognition method. Tissue samples from a tissue bank were analyzed on a nuclear magnetic resonance (NMR) spectrometer. A total of 69 samples were studied. The samples included a wide variety of malignant and benign etiologies. Using 45 samples, we initially created a model which was able to predict if a certain spectrum originates from benign or malignant lymph nodes using a pattern-recognition technique which takes into account the entire magnetic spectrum rather than single peaks alone. The remaining 24 samples were blindly loaded in the model to assess its performance. We obtained an excellent accuracy in differentiating benign and malignant lymphadenopathy using the model. It correctly differentiated as malignant or benign, in a blinded fashion, all of the malignant samples (13 of 13) and 10 out of the 11 benign samples. We thus showed that magnetic spectroscopy is able to differentiate benign from malignant causes of lymphadenopathy. Additional experiments were performed to verify that the differentiating abilities of our model were not due to differential tissue decay in between benign and malignant tissues. If future experiments demonstrate that a similar approach could be executed with standard MR imaging, this technique could be useful as a problem-solving tool when assessing lymphadenopathy in general. Alternatively, our *in-vitro* technique could also be useful to pathologists faced with indeterminate pathologies of the lymph nodes after validating our results with a larger sample size.

## Introduction

Assessment of lymphadenopathy remains one of the most challenging and often unsatisfactory issues for radiologists. Characterizing lymphadenopathy as benign or malignant using cross-sectional imaging is currently limited and a biopsy is often needed to obtain a definitive diagnosis. For example, in the neck, even in the setting of a known malignancy, up to 20% of lymph nodes that were deemed suspicious on imaging and subsequently biopsied are found to be benign [1]. Depending on the clinical setting, radiological assessment of lymphadenopathy is usually made using ultrasound (US), computerized tomography (CT), and conventional magnetic resonance imaging (MRI). These methods rely heavily on morphological characteristics, often solely based on the size of lymph nodes, which have a great degree of overlap between malignant and benign disease [2–4]. There has been recent improvement in characterization of lymphadenopathy in multiple organ systems using functional imaging techniques such as diffusion weighted imaging (DWI) and positron emission tomography (PET)-CT. However, the non-specific nature of assigning malignant or benign status remains. When assessing mediastinal lymphadenopathy, PET-CT has been shown to have an overall accuracy of 77% [5]. DWI performed only marginally better when evaluating lymphadenopathy in the neck, with an accuracy of 81% [6].

MR spectroscopy is uniquely situated to potentially improve characterization of lymph nodes because it provides information on metabolites and is not dependent on morphological traits. The spectra of some malignant and benign lymph nodes have been shown to have different characteristics in a few pathologies of the neck, axilla and breast *in-vitro* and *in-vivo* [7–10]. However it is not known if it is possible to reliably differentiate in between varied benign and malignant causes of lymphadenopathies using MRI. The aim of this study was therefore to assess if it is possible to predict whether a particular lymph node is benign or malignant using MR spectroscopy in isolation. To do this, we included in the study a wide array of pathologies in order to better replicate the potential diversity of lymphadenopathy that can be commonly encountered clinically. Additionally, while the analysis of a few single peaks is common in spectroscopy research, we alternately chose to use a pattern-recognition method which allowed us to study the entire spectrum rather than a few peaks, and potentially identify new areas of interest. An *in-vitro* approach was used, which allowed us to analyze a diverse array of pathologies using tissue banks and to determine the optimal sequence parameters to answer the clinical question.

## Materials and methods

### Specimen collection and handling

The McGill University Health Center Institutional Review Board Committee specifically approved this study. A total of 69 frozen tissue samples were included in our study. The samples were obtained from the tissue banks of the pediatric pathology and hepato-biliary departments. Of the 69 samples, 59 came from a pediatric population, 10 from an adult population. The pathological analysis was performed by the McGill pathology department; the majority of the cases were reviewed by the pediatric pathology department. Immediately after the biopsy or surgical excision, tissues that were deemed in excess by the pathologist were stored at -80 C without any processing or staining. The frozen tissues were allowed to be used for NMR experiments only after the final pathological diagnosis was confirmed by the pathology department. This was done in case more tissue was necessary to obtain the final clinical diagnosis. The time between tissue extraction and the NMR experiments ranged from 1–80 months.

In preparation for the NMR experiments, samples were thawed at room temperature, cut and fitted into a 50 μl ZrO2 Zirconia rotor (Wilmad-labglass Vineland, USA). The average weight of the samples was 30mg+/- 5mg which represented approximately 10–80% of the original frozen sample depending on the sample size. Deuterated water was used to fill the remaining space. A Teflon top insert and Kel-F cap was subsequently placed. The average time from thawing to the NMR experiment was 6 minutes. This tissue preparation method was based on the publication from Beckonert et al [11]. All experiments were performed blinded to the histopathological diagnosis and patient clinical information. Only the pathological diagnosis was made available and only at the time of the statistical multivariate analysis.

### NMR spectroscopy experiments

All of the NMR experiments were performed at the Quebec Regional Center for Spectroscopy on a Bruker Avance 400MHz (9.4Tesla) spectrometer (BrukerBioSpin, Rheinstetten, Germany). The experiments were performed at 298K using methanol for calibration. The 50 uL rotor was loaded into the spectrometer and rotated at a frequency of 5 KHz. The receiver gain was automatically adjusted before each measurement. All experiments were performed using a 4 mm ^1^H HR MAS probe head. One-dimensional proton spectra were acquired using a single RF pulse with water suppression (zgpr sequence) with 32K data points, spectral width of 6200Hz, 64 scans, and a relaxation delay of 2 seconds. The residual water was suppressed using a pre-saturation frequency pulse of 4.7 part per million (ppm). The acquisition time was 2050 msec.

### Data standardization and processing

Processing was performed in a blinded fashion. The spectra were exported and processed in Mnova software (version 10.1; Mestrelab, Santiago de la Compostella, Spain). Phase correction and baseline correction were performed manually. No zero filling or apodization functions were used. The peak alignment was performed using a recursive segment-wise peak alignment method [12]. All of the ^1^H NMR spectra were binned to 0.04ppm. The ^1^H NMR spectra were normalized using a probabilistic quotient algorithm [13]

### Statistical analysis and model

The processed data was exported to the SIMCA software (version 14.1; Umetrics Umea, Sweden). A supervised multivariate orthogonal partial least square discriminant analysis (OPLS-DA) of ^1^H NMR spectra was performed. OPLS-DA combines OPLS, a dimensionality reduction technique, with discriminant analysis, which is a form of classification. OPLS is advantageous in that class separation is achieved in the first component which allows for easier visualization of separation in between groups—this is called the predictive component. Variation which is not associated with class separation is in the subsequent or orthogonal components. In other words, these are variations which have no correlation with the studied group and are also often termed uncorrelated variation. This method aims at constructing latent variables from an n x m X-matrix of predictors (the sum values) and an n x 1 class indicator response Y vector (in our study, defined as benign or malignant). Two types of components are produced by the method: a ‘predictive’ component which is a linear combination of all the variables in X which have a maximal covariance with the response (so as to best predict Y, in our study benign or malignant); and an ‘orthogonal’ component, which summarizes the variation in X that is orthogonal to Y (therefore cannot be used for predictions). First, the data (columns of X and Y) needs to be scaled and centered. The mathematical model is:

$$X=t_{1}p_{1}^{\prime }+t_{o}P_{o}^{\prime }+E$$

$$Y=t_{1}q_{1}^{\prime }+F$$

where *t*_1_ is the n x 1 predictive component score vector and *p*_1_ and *q*_1_ are the predictive component loadings (m x 1 and 1 x 1, respectively). The model of X has a second term, the product of orthogonal scores t_o (n x k) and orthogonal loadings *P*_*o* (*m x k*), which summarizes the variation in X which is orthogonal to Y. E and F are the residual terms, and k is the number of orthogonal components. X was defined as each point along the chemical shift spectra. After binning, this yielded 390 points. To visualize the general structure of each sample and to identify any abnormalities or outliers, DmodX plot and Hotelling’s T2 plots were used [14]. Using the autofit function of the SIMCA program, the OPLS-DA training set was created with one predictive (t1) and three orthogonal components (t1o).

A total of 45 samples were used to create the training set model. After which 24 new samples were acquired at a separate time with the same parameters and blindly loaded into the OPLS-DA model to predict whether a tissue sample is benign or malignant. The sensitivity and specificity of our test was subsequently calculated based these 24 samples.

Due to the large variability in time since extraction between samples, we studied whether time since extraction had any measurable effect on our acquired spectrums. To do this, a new age-predicting OPLS-DA model with the Y response vector being time in months since tissue extraction was created using the same 45 samples. After the model was created from the training set, the same 24 samples used to differentiate in between benign and malignant tissues were loaded in the time predicting model to assess whether it was possible to predict the age of a tissue sample since extraction.

## Results

A total of 69 samples were studied, 31 were benign etiologies and 38 were malignant. The summary of the studied pathologies can be found in Table 1.

**Table 1:** Summary of the studied pathologies.

Benign	Malignant
• Reactive not otherwise specified (14) • Reactive lymphoid hyperplasia (3) • Tuberculosis (1) • Toxoplasmosis (2) • Granulomatous lymphadenopathy (2) • Folicular paracortical hyperplasia (2) • PTGC (4) • Neurofibroma (2) • Histoplasmosis (1)	• Lymphomas (Hodgkin’s, B-cell, Burkitt) (11) • Neuroblastoma (8) • Leukemias (2) • Rhabdomyosarcoma (2) • Papillary thyroid carcinoma (2) • Wilms tumors (4) • Ewings sarcoma (6) • Osteosarcoma (1) • Colorectal cancer (2)
31 cases	38 cases

**Fig 1:**
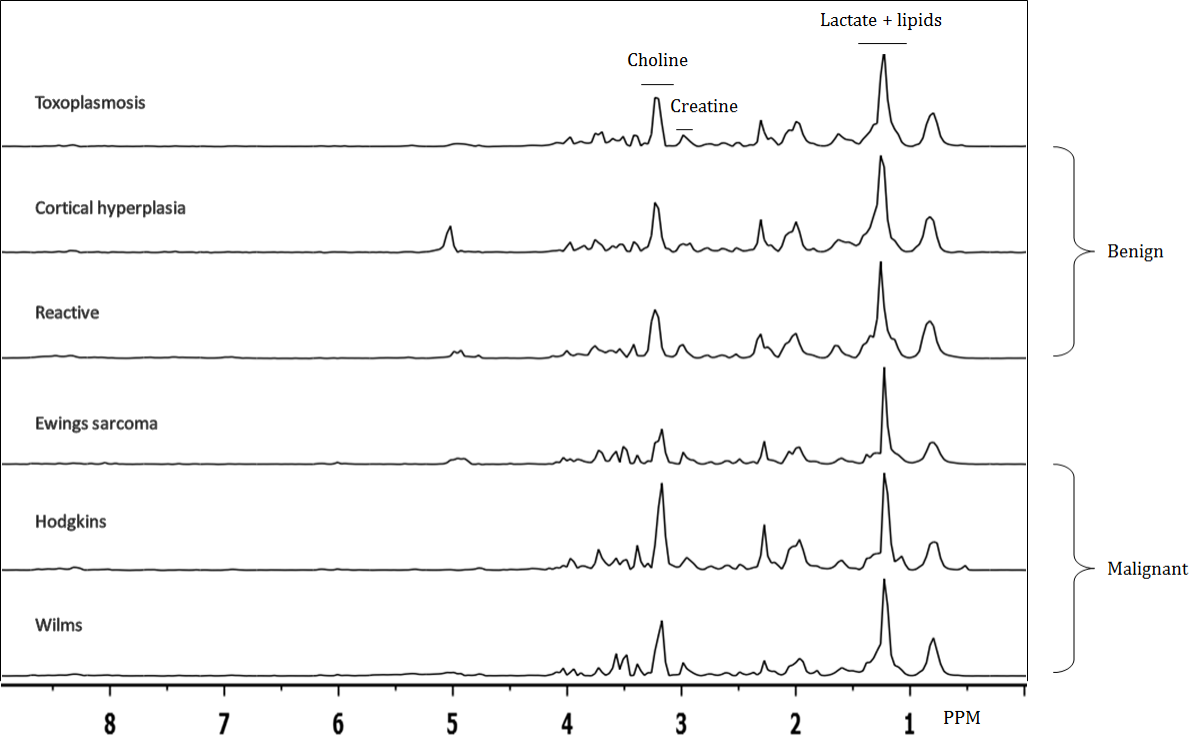
Three representative benign and malignant spectra. On visual inspection no particular peaks or patterns can be identified that predict benign or malignant character of the lymph nodes. In particular, lactate, choline and creatine peaks, which are commonly used in clinical radiology, do not give obvious predictive information.

Upon visual inspection of benign and malignant spectra, the general spectral features appeared similar. The predominant peak in the spectra was in the 1–1.5ppm range, which is a composite of multiple lipids and lactate. Examination of the spectra showed a poor spectral resolution which was expected given the binning of 0.04ppm. This did, however, make the exact identification of single peaks difficult. Representative examples of three benign and malignant spectra can be found in Fig 1.

**Fig 2:**
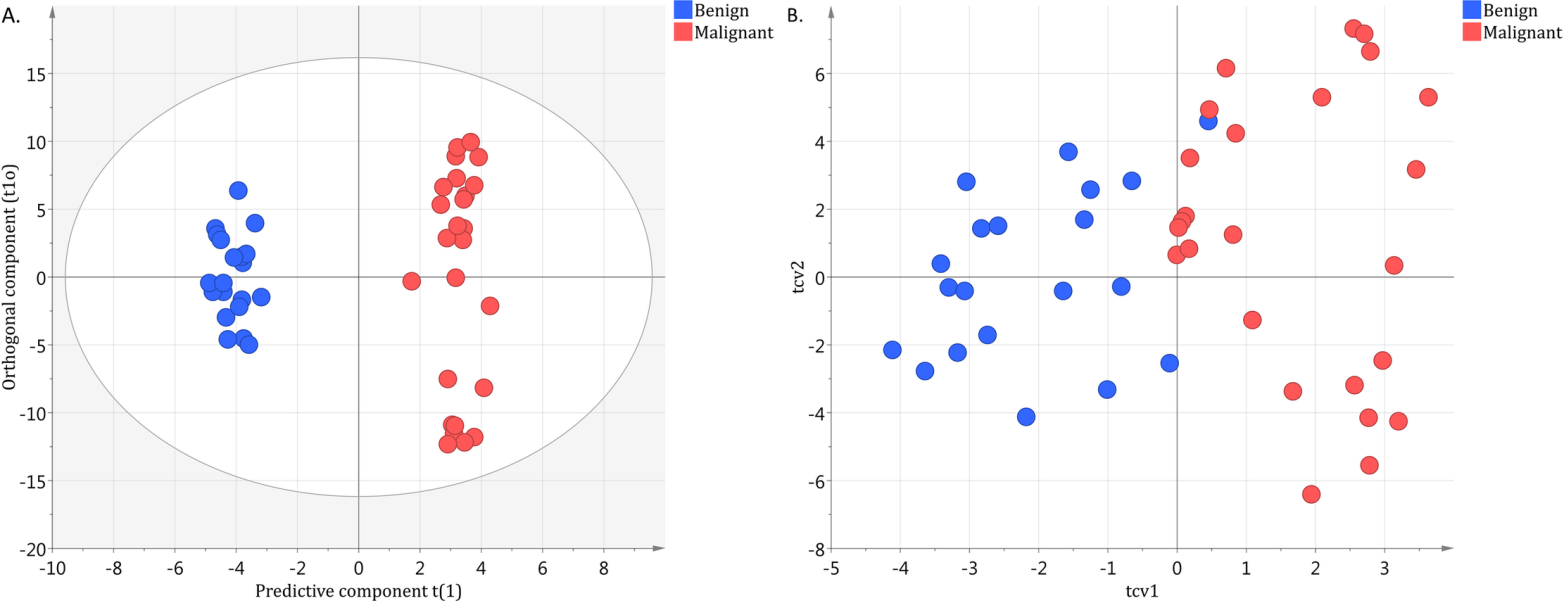
Score plot of training set differentiating benign and malignant causes of lymphadenopathy with cross-validation plot. A. t1 is the predictive component, it is used to achieve discrimination in between both groups. t1o is the orthogonal component, It is useful for understanding class variability. B. Cross-validation plot showing Tcv1 vs Tcv2 for the OPLS-DA model with three orthogonal components showing good separation of the benign and malignant groups (*R*2 = 0.96, *Q*2*Y* = 0.63, *n* = 45).

**Fig 3:**
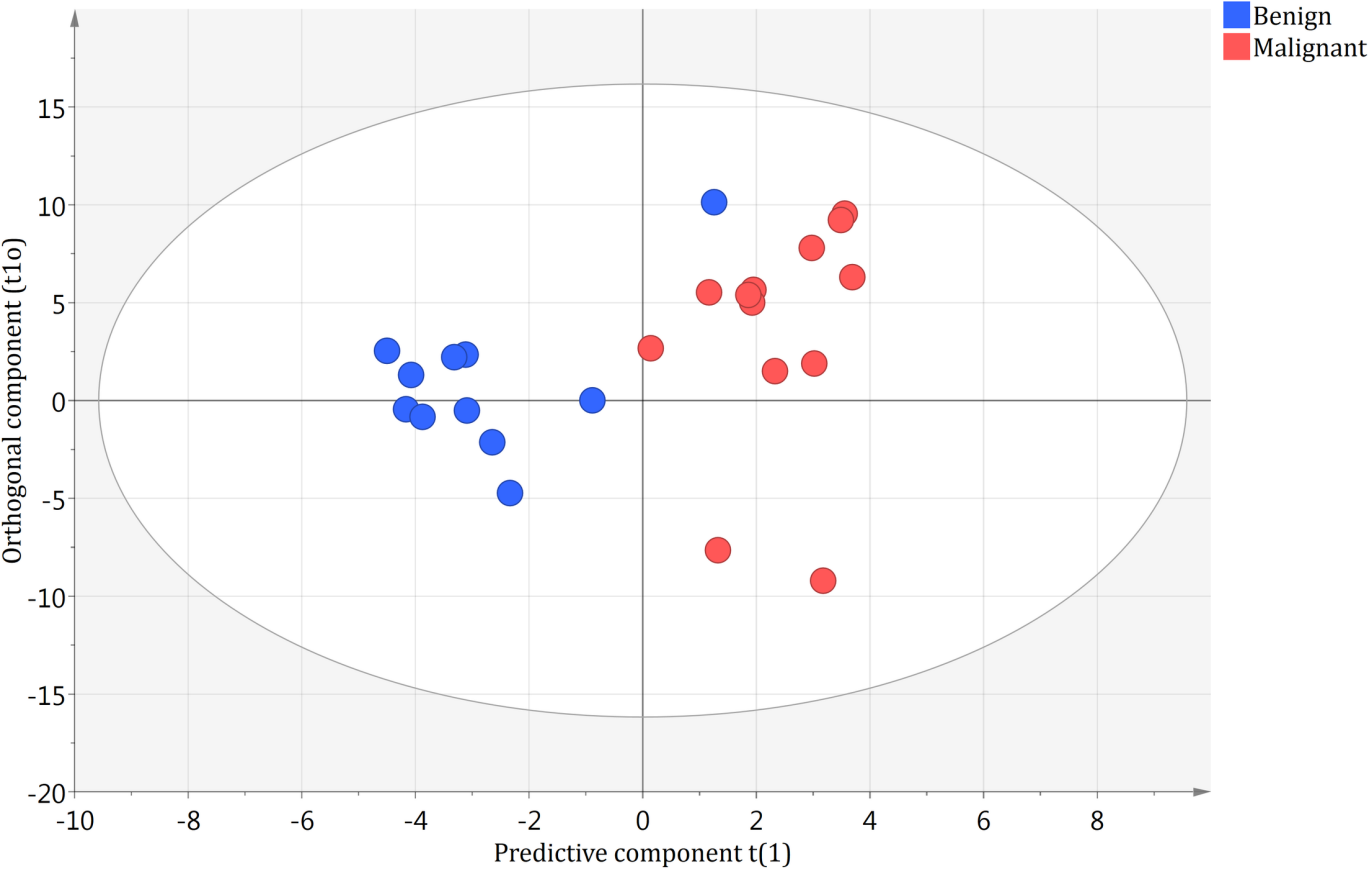
Blinded test set loaded in model predicting benign and malignant tissues. New benign and malignant samples not previously analyzed were blindly loaded into the OPLS-DA model from Fig 2, and the categories were subsequently revealed. t1 and t1o are the same as in Fig 2.

No outliers were identified using the DmodX and Hotelling’s T2 values. The OPLS-DA model showed good separation between the benign and malignant groups along the predictive component. The predictive component, called t1, has no units. It is derived from the combination of each region in the spectrum which has a high covariance with the group of interest. Each tissue sample is then given a value along t1 which is the weighted sum of each spectral region. Higher t1 values are found in malignant samples and lower t1 values are found in benign samples. The samples are clustered according to their ascribed and benign or malignant categories along the predictive component. The model showed an overall good measure of fit with an R^2^ of 0.96 and a cross-validation coefficient Q^2^Y of 0.63. These results can be visualized in Fig 2. Of the 24 blinded samples, all 13 of the malignant samples were correctly identified as malignant. These samples consisted of metastatic Ewing sarcoma (2), rhabdomyosarcoma (1), Burkitt’s lymphoma (1), neuroblastoma (3), Hodgkin’s lymphoma (3), colorectal carcinoma (2) and papillary thyroid carcinoma (1). Of the 11 benign samples, 10 were correctly identified as benign and consisted of reactive lymphadenopathy (6), lymphoid hyperplasia (1), toxoplasmosis (1), histiocytoma (1) and follicular paracortical hyperplasia (1). The single lymph node that was incorrectly identified by the model as malignant when it was in fact benign was a case of non-infectious granulomatous lymphadenopathy. These results from our test sample give a sensitivity of 100% and specificity of 91% of our model in differentiating benign from malignant lymphadenopathy using NMR spectroscopy (Fig 3).

**Fig 4:**
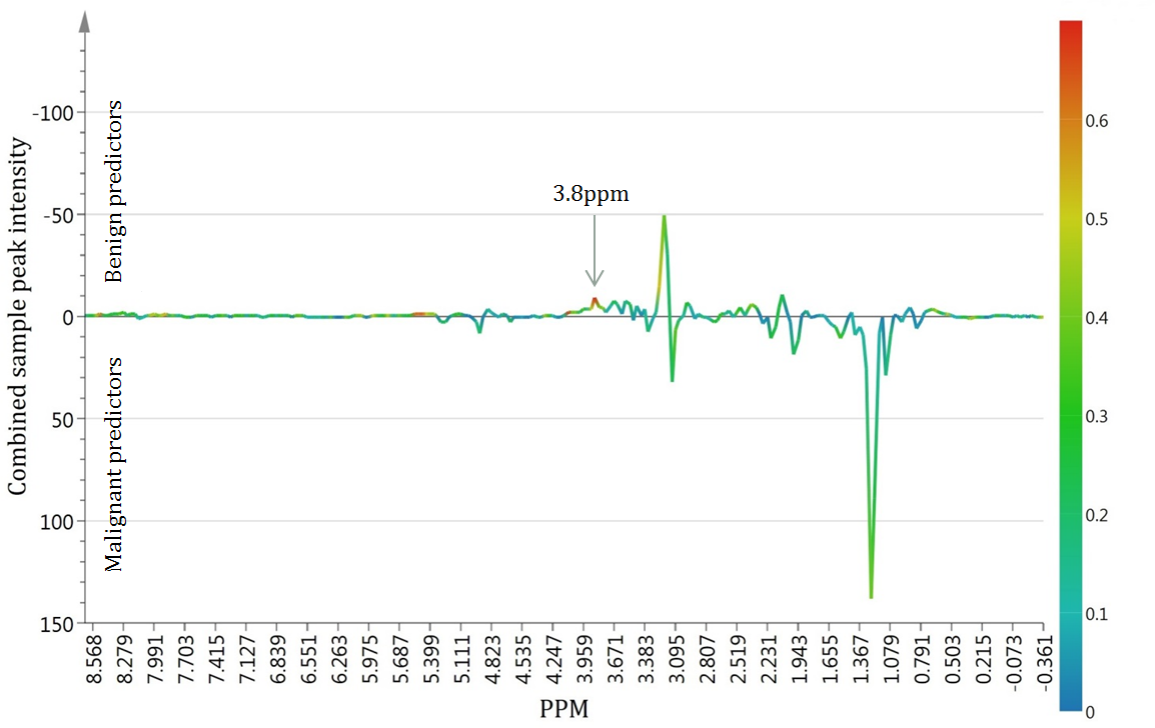
Correlation between ppm regions and the predictive component. The average of all of the studied spectra is represented along the ppm axis. This graph allows visualization of the correlation between each ppm region and the predictive t1 score. The orientation of the peak with regards to the axis indicates if they are benign predictors (above the x axis) or malignant predictors (below the x axis). Color coding corresponds to the relative weight of each peak in predicting benign vs. malignant classes. A red color indicates peaks that have a high predictive value while a blue color indicates one that has a lower predictive value. Note the peak with the highest predictive value is found at 3.8ppm.

The predictive value of each ppm region was assessed by examining the relationship of X (ppm) to the first component of Y (the predictive component). The peak that held the greatest predictive value was found at 3.8ppm and was strongly associated with a benign diagnosis. The relationship of each ppm region with the predictive component is represented in a graph, shown in Fig 4. From this graph, it can be observed that important information is present in peaks other than the most routinely used peaks in clinical MR spectroscopy (such as lactate or choline).

**Fig 5:**
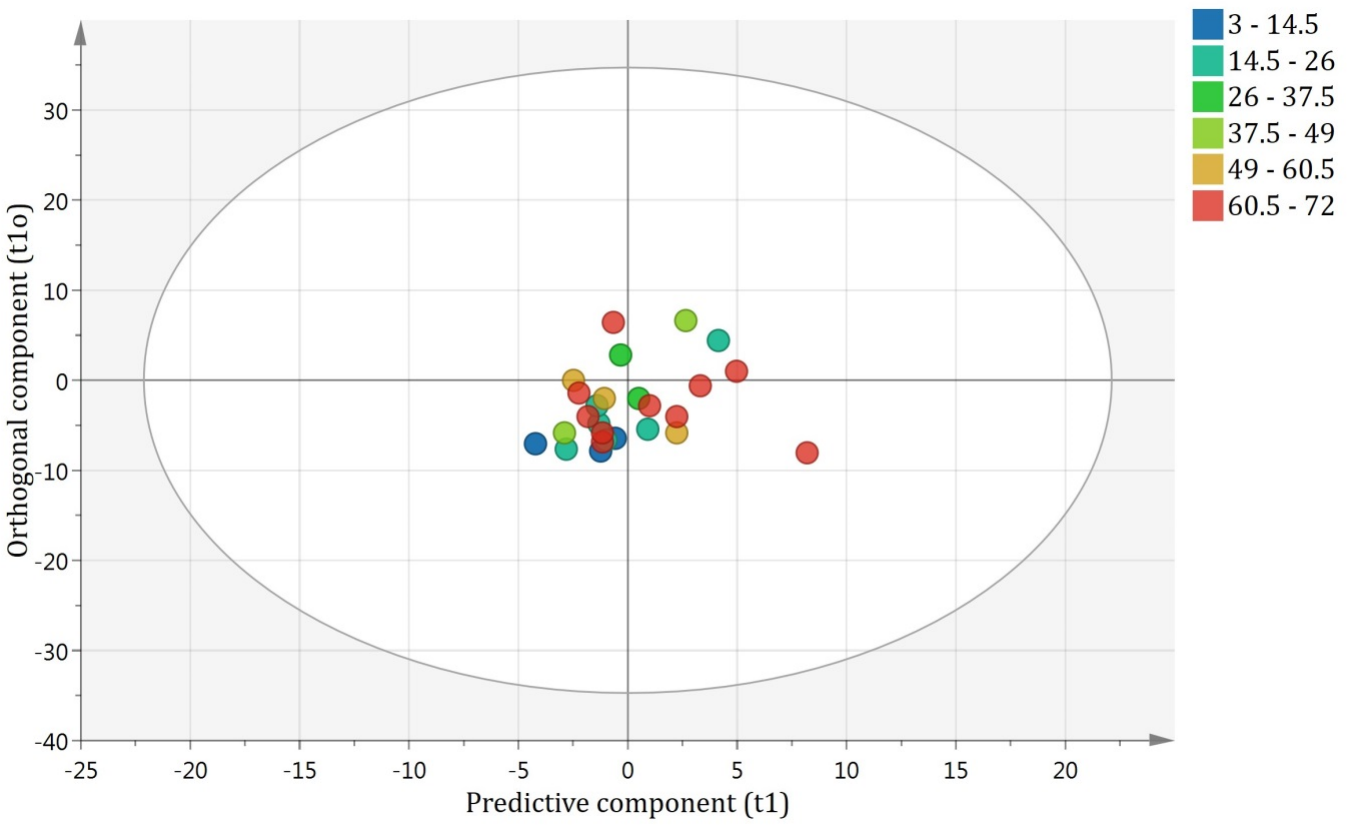
Blinded test set loaded in model predicting age of tissue. Time since collection is expressed in months. There is no separation of the samples which indicates that the age of the sample has no predictable effect of the spectrum.

For the age-predicting OPLS-DA model, lower t1 values were defined as more recent time since extraction and higher t1 values were defined as longer time since extraction. The model did not demonstrate separation abilities according to sample age along the predictive component. The 24 blinded samples did not show any separation according to age and the OPLS-DA model based on time since extraction held no predictive ability (Fig 5).

## Discussion

Correct assessment of lymphadenopathy can be one of the most challenging issues for treating physicians and radiologists. Differentiating benign and malignant lymphadenopathy remains difficult using CT and conventional MRI due to the fact that they rely heavily on morphological and macroscopic characteristics [2]. US, CT, MRI and PET scan are all used in certain settings to help characterize lymphadenopathy, however their individual performance remains disappointing even when using more advanced techniques such as DWI or contrast enhanced MRI [6, 15]. Often, a biopsy is ultimately needed to assess lymphadenopathy. In other cases, progression over time is used to differentiate benign from malignant etiologies at the expense of potential earlier treatment.

We assessed whether MR spectroscopy can differentiate benign from malignant causes of lymphadenopathy *in-vitro*. To our knowledge, our study is the first *in-vitro* MR spectroscopy experiment to focus on the differentiation of diverse benign and malignant pathologies. There were multiple advantages to the *in-vitro* approach. First, since only a small amount of tissue was necessary, samples from tissue banks could be used. Using tissue banks made it possible to study a large variety of different pathologies but also multiple samples of the same pathology, allowing us to include in the study the heterogeneous properties of similar pathologies. Secondly, no localizer or other sequences were necessary and the acquisition times were shorter, making the study of many samples relatively quick. Multiple spectroscopy sequences could be run on the same samples, thereby optimizing sequence parameters to answer the research question at hand. Lastly *in-vivo* experiments require a tissue sample to attain a definitive pathological diagnosis, however, it may not always be ethical to perform a biopsy, for example when faced with a lesion which is suspected to be benign which ultimately limits the extent of pathologies which can be studied *in-vivo* since a definitive pathological diagnosis is required.

In addition, unlike most MR spectroscopy research we studied the entire spectra as a whole rather than focusing on the analysis of a few distinctive peaks and their ratios—most commonly choline, lactate and creatine [7, 9, 16–18]. This emphasis on single peaks usually comes at the expense of losing information contained in other smaller peaks, which are deliberately removed by lengthening the echo time or are simply ignored. This results in a loss of valuable signal and information, as many of these peaks have been shown to contain important clinical information [19–21]. Analyzing the spectra as a whole is routinely used to assess sample purity in chemistry and nutritional sciences [22]. We opted to use a partial least square regression method to assess the entire spectra, which is of particular interest because it allows for a multivariate analysis of data that has numerous and noisy correlated predictors [23]. OPLS was ultimately used because class separation can be achieved in one axis which is easier for visualization. We built a regression analysis model that identified spectral components that can successfully categorize tissues as being benign or malignant. This model was able to correctly differentiate between 20 benign and 25 malignant samples. In creating this model, the alogirthm was not blinded to the histology of these samples, but actively looked for differences that would explain the studied categories, in our case benign vs. malignant. Cross validation yielded a good measure of fit R^2^ 0.96 and cross-validation coefficient Q^2^Y 0.63 suggesting that the discriminatory capabilities of the model are not due to over fitting. In order to validate the performance of the model, we loaded 24 subsequently acquired samples blindly into the model which predicted whether they were benign or malignant based on the information acquired from the prior 45 samples. The program correctly identified all of the malignant tissues and 10 out of 11 benign tissues giving a sensitivity of 100% and specificity of 91%.

In order to assess if the time since tissue extraction or the time each tissues spent at -80C had any measurable effect with our current acquisition parameters on the NMR spectrums, we created another regression analysis model that identified spectral components with predictive abilities according to tissue age. After creating the age-predicting model we found that there was no separation of samples according to age.

This indicates that with our current acquisition parameters, metabolic changes which are presumed to occur in conjunction with an increasing time since tissue extraction have no predictable effect on our acquired spectra. Therefore, we can assume that they do not play a role in our model’s ability to differentiate between benign and malignant samples.

Our study has a number of limitations. Perhaps the most important one is that our experiments were performed *in-vitro* on a 9.4 tesla scanner and therefore it is difficult to know whether our results may be extrapolated to *in-vivo* clinical MRI studies. However, several factors lead us to believe that our *in-vitro* results may be comparable to *in-vivo*. First, we performed very rapid acquisitions in order to keep our signal-to-noise within the realm of clinical MRI scanners. The spectral resolution we achieved is similar to that obtained routinely in many clinical 3T MRI even if much higher spectral resolution and signal to noise have been attained in some instances [24–26]. Second, in a study by Tugnoli et al. [27] on meningiomas, the same MR spectrometer that was used in the present study (Bruker Avance 400, 9.4T) was used to analyze the *in-vitro* samples. When Tugnoli et al. compared *in-vitro* experiments to *in-vivo* experiments performed on a 3T MRI to better identify metabolic peaks, they found them to be very similar when using the same sequence used in our study. Finally, in our analysis, we found that the single peak with the greatest predictive value for benign samples was situated at 3.8ppm. Incidentally, this same peak has been reported *in-vivo*, in MRI studies of the CNS as helpful in differentiating tuberculomas and some meningiomas from primary malignancies and metastases [28, 29]. The fact that our *in-vitro* experiments independently obtained similar results as *in-vivo* MRI experiments further suggests that our results may be attainable *in-vivo*.

Another potential limitation of our research is that only a portion of the studied lymph nodes had been assessed by the pathologist and that the studied tissues did not undergo repeat histopathological diagnosis after the experiments. However this would be a concern only if the model did not function. Given that the model correctly categorized the samples in a blinded fashion in nearly all cases, this suggests that at least the vast majority of analyzed tissues matched the categories ascribed by the pathologist. This is probably due to the small size of the studied tissues and to the fact that enlarged lymph nodes tend to be involved in their entirety by the same pathological process.

In our study we were able to demonstrate that the automated assessment of the spectra as a whole—contrary to the conventional approach which focuses on absolute values of a few or single metabolites—allows for excellent differentiation between benign and malignant lymphadenopathy *in-vitro*. In addition to being able to evaluate the entire spectra in a single step, this method of using a model appears to far exceed the subjective abilities of an individual reader. In future experiments, pre-surgical *in-vivo* spectra will be compared to postsurgical *in-vitro* spectra and pathology. The goal will be to fine tune our model in order to match it to the clinically acquired spectra and confirm the accuracy of the model in an *in-vivo* setting. The potential benefits of our approach are multiple and far-reaching. For example, after validating our results with a larger sample size, our *in-vitro* technique could be useful to pathologists when evaluating lymphadenopathy. Additionally, if future experiments demonstrate that this method could be executed using standard MRI, this technique could serve as a problem-solving tool when faced with lymphadenopathy in general.

## Conclusion

Lymphadenopathy remains a common conundrum for radiologists and treating physicians alike. We have demonstrated that MR spectroscopy can be used to differentiate between benign and malignant etiologies among a diverse group of pathologies *in-vitro*, with excellent accuracy using an automated analysis of the entire spectra.
